# Enhancement in the efficiency of heat recovery in a Williamson hybrid nanofluid over a vertically thin needle with entropy generation

**DOI:** 10.1016/j.heliyon.2023.e17665

**Published:** 2023-06-26

**Authors:** Muhammad Naveed Khan, Shafiq Ahmad, Zhentao Wang, Bandar M. Fadhl, Kashif Irshad, Sayed M. Eldin, Amjad Ali Pasha, Mohammed K. Al Mesfer, Mohd Danish

**Affiliations:** a School of Energy and Power Engineering Jiangsu University, Zhenjiang, 212013, China; b Department of Mathematics, Quaid-I-Azam University 45320, Islamabad, 44000, Pakistan; c Mechanical Engineering Department, College of Engineering and Islamic Architecture, Umm Al-Qura University, P. O. Box 5555, Makkah, 21955, Saudi Arabia; d Interdisciplinary Research Centre for Renewable Energy and Power System (IRC-REPS), Research Institute, King Fahd University of Petroleum and Minerals (KFUPM), Dhahran, 31261, Saudi Arabia; e Center of Research, Faculty of Engineering, Future University in Egypt New Cairo 11835, Egypt; f Aerospace Engineering Department, King Abdulaziz University, Jeddah, 21589, Saudi Arabia; g Chemical Engineering Department, College of Engineering, King Khalid University, Abha, Saudi Arabia

**Keywords:** Vertical thin needle, Entropy generation, Williamson hybrid nanofluid, Magnetic field, Homogeneous-heterogeneous reaction, Thermal radiation

## Abstract

The purpose of the present research is to conduct an examination of entropy generation in a 2D magneto Williamson hybrid nanofluid flow that contains cobalt ferrite and titanium oxide nanoparticles and undergoes surface-catalyzed reactions through a thin vertical needle. The consequences of joule heating and viscous dissipation are considered to elaborate the features of heat transport. Further, the influence of thermal stratification, thermal radiation, and homogeneous-heterogeneous reaction is also taken into account. Through the application of appropriate similarity variables, the dimensionless system of coupled ordinary differential equations is achieved. The coupled system of equations is numerically solved by the usage of the bvp4c technique in the MATLAB algorithm. The current investigation also compared the existing outcomes with the available literature, which shows great harmony between the two. The consequences of the physical parameters are discussed graphically and with numerical data. It is worth noting that larger values of homogeneous reaction strength and the surface-catalyzed parameter diminish the concentration field. Further, the velocity distribution and their related momentum boundary layer thickness, diminishes with the enlargement of the Weissenberg parameter.

## 1 Introduction

There are many techniques used to increase the thermal conductivity of the fluids, which is beneficial for energy purposes. An advanced way to upgrade the fluid’s thermal conductivity is to suspend the nanoparticles in the base fluid, which is called nanofluids. Nanofluids have certain exclusive features in the heat transport phenomena, which make them potentially valuable in industrial and engineering applications point of view. These fluids are made by the combination of non-metallic and metallic alloys (tiny nanoparticles) with convectional fluids (ethylene glycol, water, and oils etc.). As compared to the other convectional fluids, such fluids have high capability of thermal conductivity. Thermal conductivity performs a significant role to empower the convection heat transport rate capability of the cooling or heating fluids. The size of nanoparticles no more than 100 nm, for example Metals (*Au*, *Ag*, *Cu*), metallic oxides (*Al*_2_*O*_3_, *CuO*), alloyed (*Al*70*Cu*30), carbon nanotubes (*SWCNT*, *MWCNT*, *DWCNT*), which used to form the nanoparticles. There are various fields which include the implementations of nanofluids such as microelectronics cooling, power savings, sensing, imaging, solar energy, industrial cooling, fuels, and heating of domestic utilizations. Choi [1] initially analyzed the suspensions of nanoparticles into the convectional fluid and proposed the concept of nanofluid. Some theoretical and experimental results related to the field of nanofluid are manifested in Refs. [[2], [3], [4], [5], [6], [7], [8], [9]]. With the help of the modeling, the heat transfer mechanism of nanofluid was deliberated by Mahian et al. [10]. With the participation of a stretchable curve surface, the magnetized flow behavior influenced by chemical reactions in a Casson nanofluid was scrutinized by Kumar et al. [11]. The MHD bioconvection brought on by oxytactic microorganisms swimming in a linearly heated square cavity containing porous media and Cu-water nanofluid was numerically studied by Biswas et al. [12]. Rasheed et al. [13] worked on a stretchable rotating surface to explore the thin film flow behavior along with mechanism of heat transfer through convection in a nanofluid. The natural convection heat transfer of the Tiwari-Das model nanofluid inside a square cavity with magnetic field and a thermal radiation is numerically investigated by Sreedevi and Reddy [14]. A special type of nanofluid whose have better thermal conductivity as compared to nanofluid is called hybrid nanofluid. The combination of two or more nanoparticles with the traditional fluid yields the hybrid nanofluid. Hybrid nanofluids are extensively exploited in nanotechnological applications. The heat exchanges, radiators, electric cooling system are some well-known heat transport applications. Hybrid nanofluids have exceptional thermo-physical features to controlling the heat transport rate which is compatible with different engineering and mechanical industries. The heat transport and pressure drop features of hybrid (${Al}_{2}O_{3}–Cu/H_{2}O$) nanofluid across the uniformly heated circular tube was inspected by Suresh et al. [15]. Through a stretchable surface, the consequence of Newtonian heating on the three-dimensional flow phenomenon of a hybrid nanofluid was disclosed by Devi [16]. The futures of the mass and heat transfer of a hybrid Williamson nanofluids including Ag/MWCNT-water nanoparticles over a rotating cylinder with MHD and Cattaneo-Christov heat flux are discussed by Sreedevi and Reddy [17]. The scrutiny of flow and heat transport on radiative hybrid nanofluid (${Al}_{2}O_{3}–Cu/water$) with magnetic impact towards the stretching cylinder was ascertained by Maskeen et al. [18]. Ahmad et al. [19] observed the heat transfer features of micropolar hybrid nanofluid with the consideration of a moving thin needle. Some latest observations for simple and hybrid nanofluid flow are given in Refs. [[20], [21], [22], [23], [24], [25], [26], [27], [28]].

Thermal radiation is a very vital and interesting topic for the researcher due to their industrials and engineering applications. Thermal radiation is produced when heat formed by the movement of charges in the materials is transferred to electromagnetic radiation. All the matter which has temperature greater than zero is emitted thermal radiation. The radiative heat transfer plays a vital role in many engineering and industrials applications like solar power technology, combustion energy processes, nuclear power plant, and design of reliable equipment, gas turbines, missiles, and space vehicles. With the significance of the thermal radiation, the boundary layer mixed convective flow mechanism through a vertical plate with porous medium was detected by Bakier [29]. Khan et al. [30] explored the impact of nonlinear thermal radiation on magneto Williamson fluid involving titanium alloy particles over a thin needle. The impact of thermal radiation on a gyrotactic mixed bioconvection nanofluid flow via a vertical thin moving needle with varying Prandtl number is investigated numerically by Song et al. [31]. Reddy and Chamkha [32] concentrated on the investigation of the heat and mass transfer features over a stretching sheet implanted in porous medium with thermal radiation and non-uniform heat source/sink. With the contribution of activation energy, Muhammad et al. [33] inspected the three-dimensional flow mechanism influenced by nonlinear thermal radiation in an Eyring-Powell fluid subject to a Riga plate. The unsteady MHD Maxwell nanofluid flow via an inclined permeable stretching surface embedded in a porous medium was explored by Patil et al. [34] in the presence of thermal radiation. Recently, many researchers discuss the significant of thermal radiation (see refs. [[35], [36], [37], [38], [39], [40]]).

In heat and mass transfer analysis, the stratification impact has a vital role, and it has been extensively researched. The reasons behind the fluid’s stratification include existence of various fluid densities, variation of temperature, and concentration difference. The combined analysis of heat and mass transfer mechanisms yield the double stratification effects. Gravity causes differences in density, which is essential for the dynamics mixing of heterogeneous fluid. Thermal stratification reduces the mixing of oxygen with bottom water in the reservoirs to become the water anoxic with the action of biological processes. Higher energy efficiency can be accomplished in solar engineering by better stratification. Stratification also regulates the temperature and concentration difference between hydrogen and oxygen to prevent the growth rate of numerous species. Chen and Eichhorn [41] studied analytically natural convection flow of thermal stratified fluid subject to isothermal infinite plate. The heat and mass transfer characteristics of steady and unsteady situations of nanofluid flow over a stretched surface embedded in a porous medium with double stratification and thermal radiation are examined by Reddy and Sreedevi [42]. The boundary layer flow phenomenon and heat transfer mechanism with double stratification and thermophoresis impacts through a vertical plate was ascertained by Ibrahim and Makinde [43]. Gopal et al. [44] explored the radiative flow behavior of a Carreau nanofluid with the significance of the magnetic field subject to a permeable cylinder. With the collaboration of two rotatory disks, the flow problem of hybrid nanofluid with the analysis of heat transfer influenced by thermal stratification was numerically and statistically scrutinized by Ramzan et al. [45]. Ahmad et al. [46] worked on a stretchable exponential surface to inspect the significance of the stratification on the mechanism of heat transport and flow phenomenon developed in a hybrid micropolar nanofluid.

Overhead real-world investigation exposed that no attempt has been made to scrutinize the characteristics of entropy generation and stratification phenomena for Williamson hybrid nanofluid with homogeneous-heterogeneous reaction through a vertical thin needle. To determine the characteristics of heat transfer, the effects of viscous dissipation along with joule heating are also taken into account. The mathematical model is transferred into the suitable system of couple ODEs. The effective technique of Bvp4c is implemented to numerically figure out the nonlinear system of coupled equations. The graphical and tabulated results are obtained for various parameters to see the behavior of fluid flow and heat transport rate.

## 2 Modeling of the problem

**Fig. 1:**
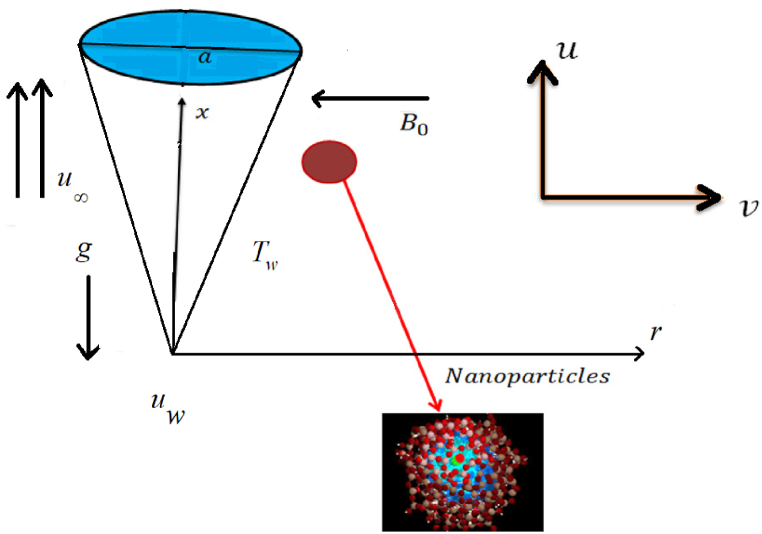
Physical description of the flow field.

In this problem we consider steady state, incompressible, 2D Williamson hybrid nanofluid flow across the vertical porous, thin needle with the impact of entropy generation and homogeneous-heterogeneous reaction. The energy equations are incorporated in the presence of thermal radiation, joule heating and viscous dissipation impact. Thermal stratification boundary conditions are imposed on the needle’s surface. In the perpendicular direction of the needle’s surface, the magnetic field is applied. The radius of the needle is taken as r=R(x). The radially and axial component is stated by r− and x− respectively, which is displayed in Fig. 1. The fluid and ambient temperature is denoted by T and $T_{\infty }$ respectively. The Williamson stress tensor is defined by [30],

(1) $$S^{*}=\tau^{*}-pI$$

(2) $$\tau^{*}=\left(1-\Gamma_{1}\gamma^{*}\right)\mu^{*}A_{1}^{*}$$

In above equations $\mu^{*}$ and $\mu_{\infty }$ are the viscosities at zero and infinity, p is the pressure, identity vector is I, and time relaxation factor is $\Gamma_{1}$. Further, $A_{1}^{*}$ is the first Rivlin-Erickson tensor and shear stress $\gamma^{*}$ are defined as,

(3) $$A_{1}^{*}=\nabla \cdot V+{\left(\nabla \cdot V\right)}^{t}$$

(4) $$\gamma^{*}=\sqrt{\frac{1}{2}\pi }\;and\;\pi =trac\left(A_{1}^{*2}\right)$$

Here, we discuss the case for $\Gamma_{1}\gamma^{*}<1$, so the above equations after using binomial expansion, we get,

(5) $$\tau^{*}=\left(1-\Gamma_{1}\gamma^{*}\right)\mu^{*}A_{1}^{*}$$

The homogeneous and heterogeneous reaction equations also defined as,

(6) $$2B^{*}+A^{*}\to 3B^{*},\text{rate}=ab^{2}k_{c}\text{,}$$

(7) $$A^{*}\to B^{*},\text{rate}=ak_{s}\text{,}$$

In the above Eqs. (6), (7), the $k_{c}$ and $k_{s}$ represents the rate constants and a and b denotes the concentration species of the chemical reactions $A^{*}$ and $B^{*}$ respectively.

The establishing equations of the flow problem by using Eqs (1), (2), (3), (4), (5), (6), (7). with above-mentioned physical assumptions and boundary layer approximation have the following expressions [30, 31],

(8) $$\frac{\partial \left(ru\right)}{\partial x}+r\frac{\partial v}{\partial r}=-v\text{,}$$

(9) $$v\left(\frac{\partial u}{\partial r}\right)=-u\left(\frac{\partial u}{\partial x}\right)+\frac{\mu_{hnf}}{\rho_{hnf}}\left(\sqrt{2}\Gamma_{1}\frac{\partial u}{\partial r}\frac{\partial^{2}u}{\partial r^{2}}+\frac{1}{r}\frac{\partial }{\partial r}\left(r\frac{\partial u}{\partial r}\right)+\frac{\Gamma_{1}}{\sqrt{2}r}{\left(\frac{\partial u}{\partial r}\right)}^{2}\right)-\frac{\sigma_{hnf}{B_{0}}^{2}}{\rho_{hnf}}u+\left(g\left[\beta_{hnf}\left(T-T_{\infty }\right)\right]-\frac{\mu_{hnf}}{\rho_{hnf}}\frac{1}{K^{*}}u\right)\text{,}$$

(10) $$v\left(\frac{\partial T}{\partial r}\right)=-u\left(\frac{\partial T}{\partial x}\right)+\frac{\alpha_{hnf}}{r}\frac{\partial }{\partial r}\left(r\frac{\partial T}{\partial r}\right)+\frac{1}{{\left(\rho C_{p}\right)}_{hnf}}\left\{\frac{16\sigma^{*}T^{3}}{3k^{*}}\left(\frac{\partial^{2}T}{\partial r^{2}}\right)+\frac{16\sigma^{*}3T^{2}}{3k^{*}}{\left(\frac{\partial T}{\partial r}\right)}^{2}\right\}+\frac{\mu_{hnf}}{{\left(\rho C_{p}\right)}_{hnf}}\left(1+\frac{\Gamma_{1}}{\sqrt{2}}\frac{\partial u}{\partial r}\right){\left(\frac{\partial u}{\partial r}\right)}^{2}+\frac{\sigma_{hnf}{B_{0}}^{2}}{{\left(\rho C_{p}\right)}_{hnf}}u^{2}\text{,}$$

(11) $$u\frac{\partial a_{1}}{\partial x}+v\frac{\partial a_{1}}{\partial r}=\frac{{D_{A}}^{*}}{r}\left(\frac{\partial a_{1}}{\partial r}+r\frac{\partial^{2}a_{1}}{\partial r^{2}}\right)-k_{1}a_{1}{b_{1}}^{2}-Sk_{s}a_{1}\text{,}$$

(12) $$u\frac{\partial b_{1}}{\partial x}+v\frac{\partial b_{1}}{\partial r}=\frac{{D_{B}}^{*}}{r}\left(\frac{\partial b_{1}}{\partial r}+r\frac{\partial^{2}b_{1}}{\partial r^{2}}\right)+k_{1}a_{1}{b_{1}}^{2}+Sk_{s}a_{1}\text{.}$$

The appropriate conditions on the boundary are given as,

(13) $$\begin{array}{l} v=0,u=u_{w},T=T_{w}=T_{0}+e_{1}x,{D_{A}}^{*}\frac{\partial a_{1}}{\partial r}=k_{s}a_{1},{D_{B}}^{*}\frac{\partial b_{1}}{\partial r}=-k_{s}a_{1},\text{at}\;r=R\left(x\right)\text{,}\\ u\to u_{w},T\to T_{\infty }=T_{0}+f_{1}x,a_{1}\to a_{0},b_{1}\to 0,\text{as}\;r\to \infty \text{.} \end{array}$$

In the above Eqs. (8), (9), (10), (11), (12), (13), u and v are the velocity components, $\rho_{hnf}$ represents the density of hybrid nanofluid’s, $\mu_{hnf}$ is hybrid nanofluid’s dynamic viscosity, $\sigma_{hnf}$ is electrical conductivity of hybrid nanofluid’s, $\alpha_{hnf}$ shows the hybrid nanofluid’s thermal diffusivity, $\left({D_{A}}^{*},{D_{B}}^{*}\right)$ is diffusion coefficient, g exhibits the gravity of fluid, $C_{p}$ denotes the specific heat capacity, $a_{0}$ is the constant, and $B_{0}$ indicates the magnetic field. Moreover, $f_{1}$ and $e_{1}$ exhibits the thermal stratification factors respectively. Table 1 gives the characteristics of the water, CoFe_2_O_4_ and TiO_2_. Meanwhile, Table 2 provides the hybrid nanofluid correlations.

**Table 1:** Nanoparticles and base fluid’s thermophysical properties.

Physical characteristics	Base fluid	Nanoparticle	
	Water $\left(H_{2}0\right)$	$TiO_{2}$	$CoFe_{2}O_{4}$
$C_{p}\left(J{\left(kgK\right)}^{-1}\right)$	4179.0	686.2	700
$\rho \left(kgm^{-3}\right)$	997.1	4250	4907
$k\left(W{\left(mK\right)}^{-1}\right)$	0.6200	8.9537	3.7
$\sigma \left(Sm^{-1}\right)$	5.50 × 10^−6^	2.38 × 10^6^	5.51 × 10^9^

**Table 2:** Relationships of hybrid nanofluid.

Characteristics	Relationships
Dynamics viscosity	$\mu_{hnf}=\frac{\mu_{f}}{{\left(1-\varphi_{hnf}\right)}^{2.5}},\varphi_{hnf}=\varphi_{1}+\varphi_{2}\text{,}$
Density	$\frac{\rho_{hnf}}{\rho_{f}}=\left(1-\varphi_{hnf}\right)+\varphi_{1}\frac{\rho_{n1}}{\rho_{f}}+\varphi_{2}\frac{\rho_{n2}}{\rho_{f}}\text{,}$
Thermal conductivity	$\frac{k_{hnf}}{k_{f}}=\left(\frac{\left(\varphi_{1}k_{n1}+\varphi_{2}k_{n2}\right){\left(\varphi_{hnf}\right)}^{-1}+2\left(\varphi_{1}k_{n1}+\varphi_{2}k_{n2}\right)+2\left(1-\varphi_{hnf}\right)k_{f}}{\left(\varphi_{1}k_{n1}+\varphi_{2}k_{n2}\right){\left(\varphi_{hnf}\right)}^{-1}+2k_{f}-\left(\varphi_{1}k_{n1}+\varphi_{2}k_{n2}\right)+\varphi_{hnf}k_{f}}\right)\text{,}$
Heat capacity	$\frac{{\left(\rho C_{p}\right)}_{hnf}}{{\left(\rho C_{p}\right)}_{f}}=\left(1-\varphi_{hnf}\right)+\frac{{\left(\rho C_{p}\right)}_{n1}}{{\left(\rho C_{p}\right)}_{f}}\varphi_{1}+\frac{{\left(\rho C_{p}\right)}_{n2}}{{\left(\rho C_{p}\right)}_{f}}\varphi_{2}\text{,}$
Electrical conductivity	$\frac{\sigma_{hnf}}{\sigma_{f}}=\left(\frac{\left(\varphi_{2}\sigma_{n2}+\varphi_{1}\sigma_{n1}\right){\left(\varphi_{hnf}\right)}^{-1}+2\left(\varphi_{1}\sigma_{n1}+\varphi_{2}\sigma_{n2}\right)+2\left(1-\varphi_{hnf}\right)\sigma_{f}}{\left(\varphi_{2}\sigma_{n2}+\sigma_{n1}\varphi_{1}\right){\left(\varphi_{hnf}\right)}^{-1}+\left(2+\varphi_{hnf}\right)\sigma_{f}-\left(\sigma_{n1}\varphi_{1}+\varphi_{2}\sigma_{n2}\right)}\right)\text{.}$

Note that, $\varphi_{1}$ and $\varphi_{2}$ denote CoFe_2_O_4_ and TiO_2_ nanoparticles, respectively, where $\varphi_{hnf}=\varphi_{1}+\varphi_{2}$. The value of solid volume fraction is taken to 0.02 (2%).

The hypothetical relation is considered as follows,

The similarity variables for the problem are stated as,

(14) $$\begin{array}{l} \eta =\frac{Ur^{2}}{x\upsilon_{f}},\theta \left(\eta \right)=\frac{T-T_{0}}{T_{w}-T_{\infty }},a_{1}=a_{0}g_{1}\left(\eta \right),b_{1}=a_{0}h_{1}\left(\eta \right)\text{,}\\ u=2Uf'\left(\eta \right),v=-\frac{\upsilon_{f}}{r}f\left(\eta \right)+\eta \frac{\upsilon_{f}}{r}f'\left(\eta \right) \end{array}$$

Using Eq. (14), the dimensionless form of the Eqs (8), (9), (10), (11), (12). is described as follows,

(15) $$\left(2\eta +8W_{e}\eta f^{\prime \prime }\right)f^{\prime \prime \prime }+\left\{2f^{\prime \prime }+6W_{e}f^{\prime \prime }f\prime \prime +A_{1}ff\prime \prime \right\}-{\left(1-\varphi_{1}-\varphi_{2}\right)}^{2.5}\frac{\sigma_{hnf}}{\sigma_{f}}Mf'-P_{m}f'+\lambda \theta =0\text{,}$$

(16) $$\frac{k_{hnf}}{k_{f}}\left(\eta \theta^{\prime \prime }+\theta \prime \right)+\frac{\mathrm{Pr}}{2}\left(A_{2}f\theta '+\frac{2\sigma_{hnf}}{\sigma_{f}}ME_{c}f\prime^{2}+\frac{2\left(1+E_{c}W_{e}f^{\prime \prime }\right)}{{\left(1-\varphi_{1}-\varphi_{2}\right)}^{2.5}}f'\prime^{2}\right)+\frac{4{\left(\theta \left(T_{r1}-1\right)+1\right)}^{2}}{3R_{d}}\left\{\left(\theta \left(T_{r1}-1\right)+1\right)\left(\eta \theta^{\prime \prime }+\frac{\theta^{\prime }}{2}\right)+3\theta \prime^{2}\left(T_{r1}-1\right)\eta \right\}=0\text{,}$$

(17) ηg1″+2g1'+Scfg1'−Sckcg1h12−ScKvsg1=0,

(18) $$\delta^{*}\left(\eta {h_{1}}^{\prime \prime }+2h_{1}\prime \right)+S_{c}f{h_{1}}^{\prime }+S_{c}k_{c}g_{1}{h_{1}}^{2}+S_{c}K_{vs}g_{1}=0\text{,}$$

The related boundary conditions (13) after transformation take the form,

(19) $$\begin{array}{l} f\left(a\right)=\frac{\varepsilon }{2}\eta ,f^{\prime }\left(a\right)=\frac{\varepsilon }{2},\theta \left(0\right)=1-S_{1},{g_{1}}^{\prime }\left(a\right)={k_{s}}^{*}g_{1}\left(a\right)\text{,}\\ \delta^{*}{h_{1}}^{\prime }\left(a\right)=-{k_{s}}^{*}g_{1}\left(a\right)\text{,}\\ f^{\prime }\left(\infty \right)\to \frac{1-\varepsilon }{2},\theta \left(\infty \right)\to 0,g_{1}\left(\infty \right)\to 1,h_{1}\left(\infty \right)\to 0\text{.} \end{array}$$

The appropriate parameters involved in the above equations are symbolized by, M represents the magnetic parameter, $S_{1}$ is the thermal stratification parameter, $W_{e}$ is Weissenberg number, $E_{c}$ is Eckert number, $K_{vs}$ is strength of heterogeneous reaction parameter, $R_{d}$ is radiation parameter, $P_{m}$ is porosity parameter, Pr is parameter and $S_{c}$ Schmidt number. Further, it is noted that if ε is stretching parameter which varies between (0<ε<1), then the fluid direction and the needle movement is same. The mathematical relation of parameters is written as in Eq. (20),

(20) $$\begin{array}{l} \mathrm{Pr}=\frac{\upsilon_{f}}{\alpha_{f}},R_{d}=\frac{k_{f}k^{*}}{4{T_{\infty }}^{3}\sigma^{*}},M=\frac{\sigma_{f}{B_{0}}^{2}}{2\rho_{f}U},W_{e}=\frac{\sqrt{2}\Gamma_{1}U^{2}r{Re}_{x}}{\upsilon_{f}x{Re}_{r}},S_{1}=\frac{f_{1}}{e_{1}},\lambda =\frac{G_{rx}}{R{e_{x}}^{2}},T_{r1}=\frac{T_{w}}{T_{\infty }}\text{,}\\ G_{rx}=\frac{\left(T_{w}-T_{\infty }\right)g\rho_{f}\beta_{T}x^{3}}{{\upsilon_{f}}^{2}\rho_{f}},{Re}_{x}=\frac{Ux}{\upsilon_{f}},{Re}_{r}=\frac{Ur}{\upsilon_{f}},\varepsilon =\frac{u_{w}}{U},{k_{s}}^{*}=\frac{k_{s}}{{D_{A}}^{*}}\sqrt{\frac{\upsilon_{f}x}{U}},k_{c}=\frac{k_{1}{a_{o}}^{2}x}{u_{w}}\text{,}\\ A_{1}={\left(1-\varphi_{1}-\varphi_{2}\right)}^{2.5}\left(\left(1-\varphi_{1}-\varphi_{2}\right)+\varphi_{1}\frac{\rho_{s1}}{\rho_{f}}+\varphi_{2}\frac{\rho_{s2}}{\rho_{f}}\right),A_{2}=\left(1-\varphi_{1}-\varphi_{2}\right)+\varphi_{1}\frac{{\left(\rho C_{p}\right)}_{s1}}{{\left(\rho C_{p}\right)}_{f}}+\varphi_{2}\frac{{\left(\rho C_{p}\right)}_{s2}}{{\left(\rho C_{p}\right)}_{f}}\text{.} \end{array}$$

If the diffusion coefficient $D_{A}^{*}$ and $D_{B}^{*}$ are equal, i.e., $\delta^{*}=1$,

(21) $$g_{1}\left(\eta \right)+h_{1}\left(\eta \right)=1\text{.}$$

Now applying the above property [21], Eqs. (17), (18). and their corresponding boundary condition take the form [22,23],

(22) ηg1″+2g1'+Scfg1'−Sckcg1(1−g1)2−ScKvsg1=0,

(23) $${g_{1}}^{\prime }\left(a\right)={k_{s}}^{*}g_{1}\left(a\right),g_{1}\left(\infty \right)\to 1\text{.}$$

## 3 Physical quantities

For the current analysis, the essential physical quantities (Skin friction coefficient, Nusselt number) have the following expressions,

(24) $$Nu_{x}=\frac{xq_{w}}{k_{f}\left(T_{w}-T_{0}\right)},C_{f}=\frac{\tau_{w}}{\rho_{f}U^{2}}\text{,}$$

Such that in Eq. (24). $\tau_{w}$ is surface shear stress and $q_{w}$ represent the heat flux at wall

(25) $$\tau_{w}=\mu_{hnf}{\left(\frac{\partial u}{\partial r}\right)}_{r=a},q_{w}={\left(-k_{hnf}\frac{\partial T}{\partial r}-\frac{16\sigma^{*}T^{3}}{3k^{*}}\frac{\partial T}{\partial r}\right)}_{r=a}$$

Using Eq. (25). in Eq. (24), the dimensionless form of skin friction and Nusselt number becomes,

(26) $$\begin{array}{l} C_{f}R{a_{x}}^{1/4}=\frac{4\mu_{hnf}}{\mu_{f}}\sqrt{a}\left(f^{\prime \prime }\left(a\right)+\frac{We}{2}f^{\prime \prime }{\left(a\right)}^{2}\right)\text{,}\\ Nu_{x}R{e_{x}}^{-1/2}=-2\sqrt{a}\theta^{\prime }\left(a\right)\left(\frac{k_{hnf}}{k_{f}}+\frac{4}{3R_{d}}{\left(\left(T_{r1}-1\right)\theta \left(a\right)+1\right)}^{3}\right)\text{.} \end{array}$$

In Eq. (26), ${Re}_{x}$ is the Reynolds number.

## 4 Entropy generation

The entropy generation is very important phenomena of irreversibility process. It measures the disorderness of the surrounding and system. Entropy generation is calculated when fully heat transfer does not occur in the system. The entropy equation is sated as,

(27) $$S^{\prime \prime }{\prime \prime }_{gen}^{\prime \prime }=\frac{k_{hnf}}{{T_{\infty }}^{2}}\left(1+\frac{16\sigma^{*}{T_{\infty }}^{3}}{3k_{f}k^{*}}\right){\left(\frac{\partial T}{\partial r}\right)}^{2}+\frac{\mu_{hnf}}{T_{\infty }}{\left(\frac{\partial u}{\partial r}\right)}^{2}\left(1+\frac{\Gamma^{*}}{\sqrt{2}}\frac{\partial u}{\partial r}\right)+\frac{\sigma_{hnf}}{T_{\infty }}{B_{0}}^{2}u^{2}+\frac{R{D_{A}}^{*}}{a_{o}}{\left(\frac{\partial a_{1}}{\partial r}\right)}^{2}+\frac{R{D_{A}}^{*}}{T_{\infty }}\left(\frac{\partial T}{\partial r}\right)\left(\frac{\partial a_{1}}{\partial r}\right)+\frac{R{D_{B}}^{*}b_{1}}{a_{0}}{\left(\frac{\partial b_{1}}{\partial r}\right)}^{2}+\frac{R{D_{B}}^{*}}{T_{\infty }}\left(\frac{\partial T}{\partial r}\right)\left(\frac{\partial b_{1}}{\partial r}\right)+\frac{\mu_{hnf}}{T_{\infty }K}u^{2}\text{,}$$

The ${\left(S^{\prime \prime }{\prime \prime }_{gen}^{\prime \prime }\right)}_{c}$ is defined as,

(28) $${\left(S^{\prime \prime }{\prime \prime }_{gen}^{\prime \prime }\right)}_{c}=\frac{4k_{f}U}{\upsilon_{f}x}$$

Using the above similarity transformation [14] in(27), (28), the non-dimensionless form of entropy generation becomes,

(29) NG=S″″gen″(S″″gen″)c=khnfkfη(Tr1−1)2θ′2(1(1+(Tr1−1)θ)2+4(1+(Tr1−1)θ)3Rd)+2Br(Tr1−1)(1+(Tr1−1)θ)(M+Pm(1−φ1−φ2)2.5)f′2+4Br(Tr1−1)ηf′′2(1+WeEcf″)(1+(Tr1−1)θ)(1−φ1−φ2)2.5+4η(Tr1−1)(L1−L2)(1+(Tr1−1)θ)θ'g1'+4η(Tr1−1)(L1+L2)(1+(Tr1−1)θ)α1g12,

In the above Eq. (29) the paraments are $T_{r1}$ is temperature ratio parameter and $B_{r}$ is the Brinkman number.

## 5 Numerical method and evidence

The numerical solutions of coupled nonlinear ordinary differential Eqs. (15), (16), (17), (18) with boundary conditions (19) are now solved using the MATLAB BVP-4c functions. Only first-order ordinary differential equations can be solved using the MATLAB BVP-4c tools. We eliminated 10^−6^ as the absolute convergence criterion and converted the third and second order differential equations to first order, and selected a fair value for $\eta_{\infty }$. As a result, we placed the first order classifications in Eqs. (30), (31), (32), (33), (34), (35),

(30) f=y(1),f′=y(2),f″=y(3),θ=y(4),θ'=y(5),g1=y(6),g1'=(7),

(31) $$yy1=\frac{1}{\left(2\eta +8W_{e}\eta y\left(3\right)\right)}\left[\begin{array}{l} -\left\{2y\left(3\right)+6W_{e}y\left(3\right)y\left(3\right)+A_{1}fy\left(3\right)y\left(1\right)\right\}+{\left(1-\varphi_{1}-\varphi_{2}\right)}^{2.5}\frac{\sigma_{hnf}}{\sigma_{f}}My\left(2\right)\\ +P_{m}y\left(2\right)-\lambda y\left(4\right) \end{array}\right]\text{,}$$

(32) $$yy2=\frac{1}{\left\{\frac{k_{hnf}\eta }{k_{f}}-\frac{4{\left(y\left(4\right)\left(T_{r1}-1\right)+1\right)}^{3}\eta }{3R_{d}}\right\}}\left\{\begin{array}{l} -\frac{\mathrm{Pr}}{2}\left(\frac{2\sigma_{hnf}}{\sigma_{f}}ME_{c}y\left(2\right)y\left(2\right)+\frac{2\left(1+E_{c}W_{e}y\left(3\right)\right)}{{\left(1-\varphi_{1}-\varphi_{2}\right)}^{2.5}}y{\left(3\right)}^{2}\right)\\ -\frac{k_{hnf}}{k_{f}}y\left(5\right)-A_{2}\frac{\mathrm{Pr}}{2}y\left(1\right)y\left(5\right)\\ -\frac{4{\left(\theta \left(T_{r1}-1\right)+1\right)}^{2}}{3R_{d}}\left\{\left(y\left(4\right)\left(T_{r1}-1\right)+1\right)\frac{y\left(5\right)}{2}+3y{\left(5\right)}^{2}\left(T_{r1}-1\right)\eta \right\} \end{array}\right\}\text{,}$$

(33) $$yy3=\frac{1}{\eta }\left\{-2y\left(7\right)-S_{c}y\left(1\right)y\left(7\right)+S_{c}k_{c}y\left(6\right){\left(1-y\left(6\right)\right)}^{2}+S_{c}K_{vs}y\left(6\right)\right\}\text{,}$$

with the conditions,

(34) $$y_{a}\left(1\right)=\frac{\varepsilon }{2}\eta ,y_{a}\left(2\right)=\frac{\varepsilon }{2},y_{a}\left(4\right)=1-S_{1},y_{a}\left(7\right)={k_{s}}^{*}y_{a}\left(6\right)\text{,}$$

(35) $$y_{\inf}\left(2\right)-\frac{1-\varepsilon }{2};\;y_{\inf}\left(4\right);\;y_{\inf}\left(6\right)\text{.}$$

**Fig. 2:**
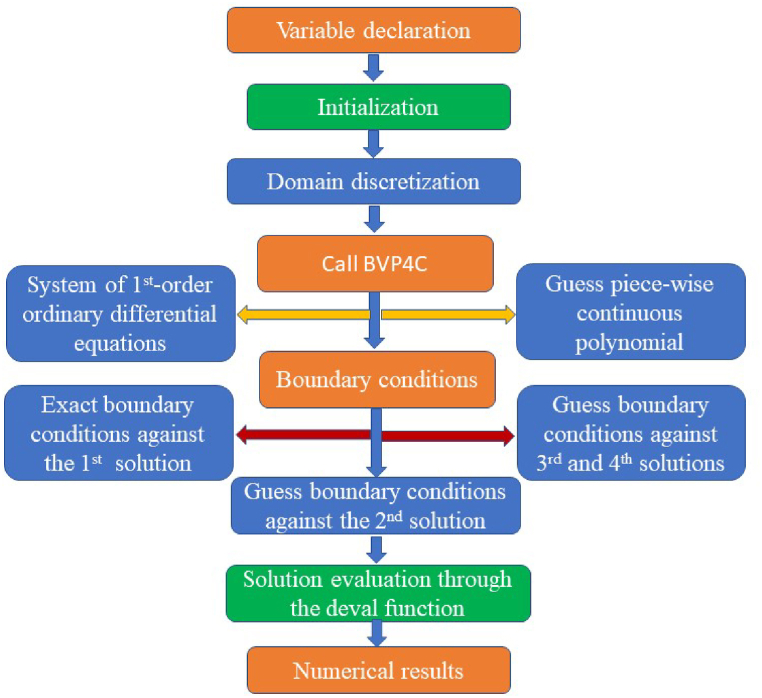
Flow chart of the considered numerical method.

This study uses the $\eta_{\infty }=6$ conclusion to achieve the asymptotic values specified in the boundary condition (19). We are sure in the accuracy and precision of the current conclusion because the relationship establishes a high level of comprehension for each value considered. The validation technique is carried out firmly to establish the validity of the current model. In this regard, a direct comparison study of $f^{\prime \prime }\left(a\right)$ is conducted with earlier research (see refs. [47, 48[47, 48]) to the distinct values of a. The numerical results see Table 3, which shows that the values of a consideration are reached with very solidagreements. In Fig. 2 the flow chart of the bvp4c method is discussed.

**Table 3:** Comparative analysis for $f^{\prime \prime }\left(a\right)$ for $M=\lambda =0=P_{m}=W_{e}$.

a	Ishak et al. [47]	Chen and Smith [48]	Present study
0.1	1.2888	1.28882	1.28881
0.01	8.49240	8.492443	8.49247
0.001	62.16370	62.163720	62.16374

## 6 Results and explanation

This section is set up to discuss the behavior of various features of flow problems (concentration, velocity, entropy generation, temperature) corresponding to the physical parameters by Sgraphically and numerically. The numerical evaluation of the nonlinear equations is carried out via an effective bvp4c methodology. This solver uses a fourth-order accurate finite difference approach to apply the 3-stage Lobatto IIIa formula. Table 4 is arranged to numerically examine the impact of numerous emerging parameters on the physical quantities of Nusselt number and skin friction coefficient.

**Table 4:** Numerical results of skin friction and Nusselt number with fixed value of λ=0.3,M=2.0,Pr=6.2**,**$\varphi_{1}=0.01,\varphi_{2}=0.03$.

$P_{m}$	$W_{e}$	a	ε	$R_{d}$	$C_{f}R{a_{x}}^{1/4}$	$Nu_{x}R{e_{x}}^{-1/2}$
0.1	0.01	0.01	0.1	0.1	1.34920	2.36301
0.2					1.35610	2.35861
0.3					1.36400	2.34171
	0.02				1.36520	2.54301
	0.03				1.56630	2.53571
	0.04				1.76740	2.52911
		0.02			1.66821	2.92301
		0.03			1.38132	2.48996
		0.04			1.08943	2.03404
			0.2		1.46821	2.32301
			0.3		1.63092	2.34692
			0.4		1.79513	2.36693
				0.3	1.26521	2.62301
				0.5	1.26522	2.59122
				0.7	1.26523	2.56943

**Fig. 3:**
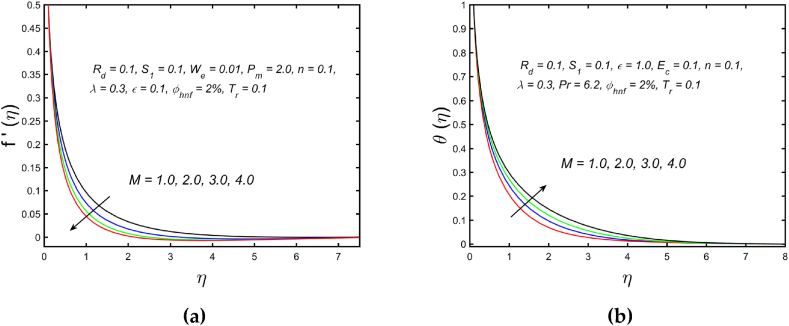
Effect of magnetic parameter on (a) velocity and (b) temperature field.

**Fig. 4:**
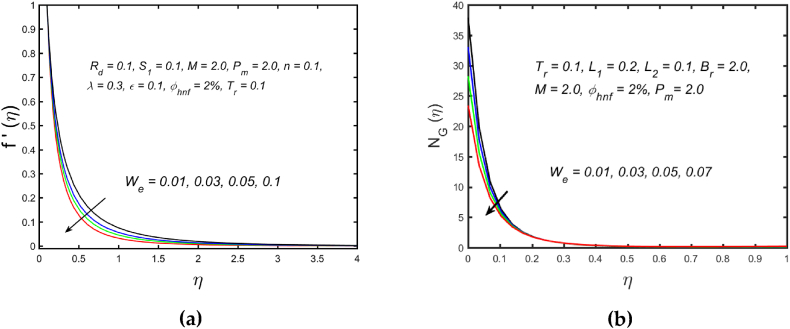
Variation of Weissenberg parameter on (a) velocity and (b) entropy generation field.

**Fig. 5:**
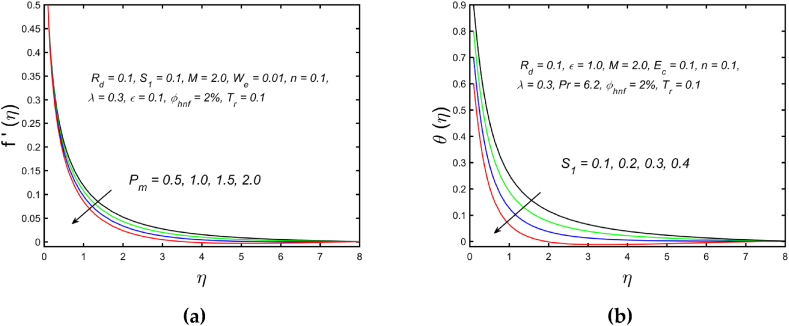
Variation of (a) porous parameter on velocity (b) thermal stratification on temperature field.

**Fig. 6:**
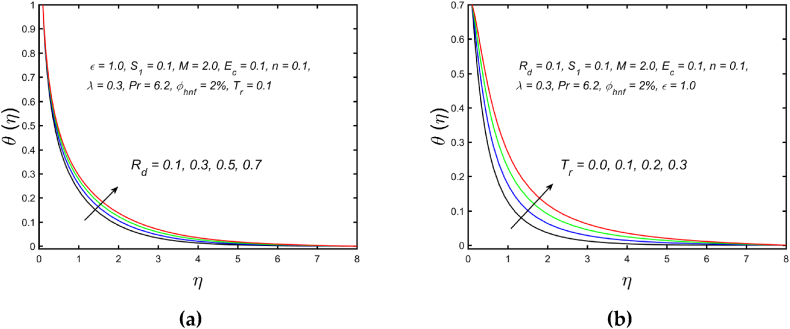
Variation of (a) radiation parameter and (b) temperature ratio parameter against temperature field.

**Fig. 7:**
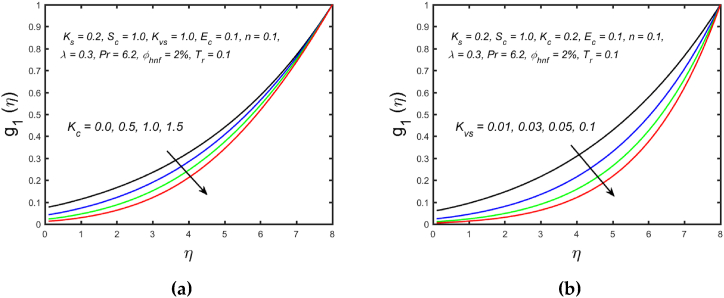
Variation of (a) homogeneous reaction strength and (b) surface catalyzed parameter on concentration field.

**Fig. 8:**
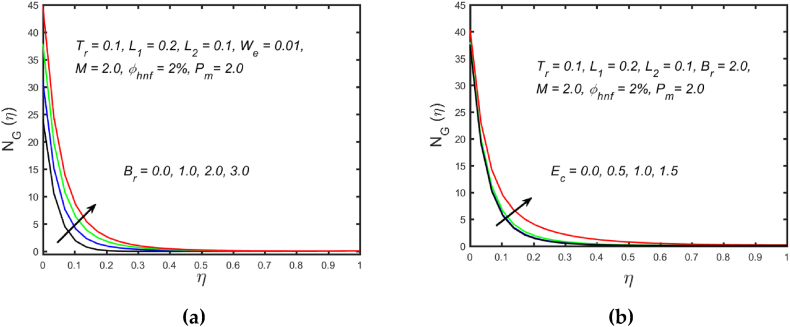
Variation of (a) Brinkman number and (b) Eckert number on entropy generation field.

Fig. 3(a) and (b) illustrate the influence of magnetic parameters on the velocity distribution and temperature distribution. These graphics reveal that the higher amount of magnetic field leads to an increment in the temperature curve but lowers the velocity distribution. Physically, with a larger magnetic parameter, a Lorentz force occurs which further develops a resistance in the movement of the fluid. Consequently, the fluid velocity slows down and fluid temperature is boosted. Fig. 4(a) captured the properties of the Weissenberg parameter on the fluid velocity. From the picture, it is clear that the velocity distribution, as well as their related momentum boundary layer thickness diminishes with the improved Weissenberg parameter. Since the relaxation to retardation time ratio is the Williamson parameter. As a result, uplifted values of Weissenberg parameter increase relaxation time. The liquid particles must take more time to restore their original path as a result fluid velocity slow down. The same behavior for entropy generation field is observed, which is depicted in Fig. 4(b). The influence of the porosity parameter on fluid velocity and thermal stratification on temperature distribution is highlighted in Fig. 5((a) and (b)). With the accelerating amount of the porosity parameter, the declining nature of the velocity profile is manifested in Fig. 5(a). Further, the augmented porosity parameter lowers the thickness of the momentum boundary layer. Physically, it is noted that greater values of porosity parameter reduce the permeability of which results in producing more resistance to the fluid, therefore the fluid velocity reduces. The upshot of thermal stratification is observed in Fig. 5(b). It is demonstrated that the larger magnitude of the thermal stratification develops a reduction in the thermal boundary layer thickness and in the temperature distribution. The reason behind this phenomenon is that the augmented thermal stratification parameter produces a decrement in the potential difference between the ambient liquid temperature and needle surface, which further causes a reduction in the corresponding thermal boundary layer thickness. The pattern of the temperature distribution corresponding to the radiation parameter and temperature ratio parameter is disclosed in Fig. 6(a) and (b) respectively. It is identified that with the improved intensity of the radiation parameter, Fig. 6(a) exhibits the inclining nature of the temperature field. Physically fluid is heated more and more with stronger estimation of radiation parameter, thus the fluid temperature enhances. The consequence of the temperature ratio parameter on the profile of the temperature is manifested in Fig. 6(b). The temperature gradient in this case is increased by a rise in the temperature ratio parameter. The response of the different values of homogeneous reaction strength and surface catalyzed parameter on the curve of the concentration is perceived in Fig. 7((a) and (b)). The concentration profile declines as homogeneous reaction and surface catalyzed parameter values increase. Fig. 8((a) and (b)) depict the nature of the entropy generation against the parameter η regarding the Brinkman and Eckert numbers. The graphical pattern explains that the Brinkman number and Eckert number increase the entropy generation profile.

## 7 Concluding remarks

In this examination, we have explored the hybrid nanofluid flow over a thin vertical needle with surface catalyzed reaction and entropy generation. Moreover, the influences of homogeneous-heterogeneous reaction, thermal stratification, and Joule heating are discussed. The transformed equation is tackled by the usage of the finite difference method in bvp4c technique. The current analysis has the following essential results.

➢ The fluid velocity reduces with magnetic parameter while temperature enhances due to Lorentz force.
➢ The Weissenberg number diminished the boundary layer thickness and entropy generation field.
➢ With the enlargement of the porosity parameter, the rate of heat transport reduces, and the drag force enhances.
➢ Radiation and temperature ratio parameter boost the temperature curve and the reverse nature are observed corresponding to the thermal stratification parameter.
➢ The homogeneous reaction strength and surface catalyzed parameter reduces the field of concentration.
➢ The entropy production field enhances with a higher estimation of Brinkman number and Eckert number.

## Author contribution statement

Muhammad Naveed Khan: Conceived and designed the experiments; Performed the experiments; Wrote the paper.

Shafiq Ahmad: Conceived and designed the experiments; Contributed reagents, materials, analysis tools or data; Wrote the paper.

Zhentao Wang: Bandar M. Fadhl: Conceived and designed the experiments; Analyzed and interpreted the data; Wrote the paper.

Kashif Irshad: Sayed M Eldin: Performed the experiments; Analyzed and interpreted the data; Wrote the paper.

Amjad Ali Pasha: Mohammed K. Al Mesfer: Analyzed and interpreted the data; Wrote the paper.

Mohd Danish: Contributed reagents, materials, analysis tools or data; Wrote the paper.

## Data availability statement

Data will be made available on request.

## Declaration of competing interest

The authors declare that they have no known competing financial interests or personal relationships that could have appeared to influence the work reported in this paper
